# Favorable Outcomes after Implantation of Biodegradable Polymer Coated Sirolimus-Eluting Stents in Diabetic Population: Results from INDOLIMUS-G Diabetic Registry

**DOI:** 10.1155/2015/265670

**Published:** 2015-09-02

**Authors:** Anurag Polavarapu, Raghava Sarma Polavarapu, Jayesh Prajapati, Asif Raheem, Kamlesh Thakkar, Shivani Kothari, Ashok Thakkar

**Affiliations:** ^1^Lalitha Super Speciality Hospital (P) Ltd., Heart and Brain Centre, Kothapet, Guntur, Andhra Pradesh 522001, India; ^2^Apollo Hospitals, Plot No. 1 A, Bhat GIDC Estate, Gandhinagar, Gujarat 382428, India; ^3^Yashfeen Cardiac Hospital, Navsari, Gujarat 396445, India; ^4^Lions Sterling Super Specialty Hospital, Mehsana, Gujarat 384001, India; ^5^Department of Clinical Trials, Sahajanand Medical Technologies Pvt. Ltd., Surat, Gujarat 395004, India

## Abstract

*Objective*. The main aim is to evaluate safety, efficacy, and clinical performance of the Indolimus (Sahajanand Medical Technologies Pvt. Ltd., Surat, India) sirolimus-eluting stent in high-risk diabetic population with complex lesions. *Methods*. It was a multicentre, retrospective, non-randomized, single-arm study, which enrolled 372 diabetic patients treated with Indolimus. The primary endpoint of the study was major adverse cardiac events (MACE), which is a composite of cardiac death, target lesion revascularization (TLR), target vessel revascularization (TVR), myocardial infarction (MI), and stent thrombosis (ST). The clinical follow-ups were scheduled at 30 days, 6 months, and 9 months. *Results*. The mean age of the enrolled patients was 53.4 ± 10.2 years. A total of 437 lesions were intervened successfully with 483 stents (1.1 ± 0.3 per lesion). There were 256 (68.8%) male patients. Hypertension and totally occluded lesions were found in 202 (54.3%) and 45 (10.3%) patients, respectively. The incidence of MACE at 30 days, 6 months and 9 months was 0 (0%), 6 (1.6%), and 8 (2.2%), respectively. The event-free survival at 9-month follow-up by Kaplan Meier method was found to be 97.8%. *Conclusion*. The use of biodegradable polymer coated sirolimus-eluting stent is associated with favorable outcomes. The results demonstrated in our study depict its safety and efficacy in diabetic population.

## 1. Introduction

Diabetes mellitus experiences a rampant growth and is currently affecting more than 150 million people worldwide. The onset of diabetes heralds the beginning of the macrovascular complications of our body. Cardiovascular disease accounts for about 80% of the deaths in diabetic populations [1]. The correlation between diabetes and cardiovascular diseases is still nebulous and is presumed to be related to hyperglycemia, hyperinsulinemia, altered lipid metabolism, hypercoagulability, and inflammation [2, 3]. All these propitious changes accelerate atherosclerotic lesion formation causing cardiovascular morbidities. The consequences of cardiovascular intervention in diabetic population are a little less promising. The malefactor behind this is more diffused, deep-rooted, and advanced nature of coronary artery disease in such diabetic individuals [4]. Moreover, the anatomy of coronary arteries involves small vessels and long lesions [5, 6]. The chances of platelet aggregation and thrombotic events are more in diabetics than in nondiabetic individuals [7]. These pose a challenge against treatment with percutaneous coronary intervention (PCI) due to more repetitive restenosis, late luminal loss, and stent thrombosis. But much improvements have been seen in medical management by PCI, if we compare 1-year mortality rates of Bypass Angioplasty Revascularization Investigation (BARI) with Arterial Revascularization Therapy Study (ARTS) [2, 8, 9]. But still there are multiple opinions and dilemmas regarding favorable outcomes of biodegradable polymer coated sirolimus-eluting stents in diabetic population.

Thus, the main aim of our study is to demonstrate safety and efficacy of biodegradable polymer coated sirolimus-eluting stents in diabetic population.

## 2. Methods

### 2.1. Study Design and Patient Population

This was a retrospective, single-arm, non-randomized, multicentre registry involving diabetic patients treated with Indolimus sirolimus-eluting stents (Sahajanand Medical Technologies Pvt. Ltd.) from June 2012 to May 2014. The ethical approval was obtained from institutional ethics committee. Written informed consent was obtained from all the patients enrolled in the study or from their legally authorized representative. The study was conducted in accordance with the principle of good clinical practice and Declaration of Helsinki.

#### 2.1.1. Inclusion Criteria

Patients were included if they were at least 18 years of age, had diabetes mellitus according to World Health Organization Report [10], and presented with stable or unstable angina or myocardial ischemia or acute or recent myocardial infarction.

#### 2.1.2. Exclusion Criteria

Patients were excluded (1) if they had known allergy to aspirin, clopidogrel, cobalt-chromium, heparin, ticlopidine, sirolimus, and polymers or (2) if the patient had impaired glucose tolerance without pharmacologic treatment, transient hyperglycemia, or gestational diabetes.

### 2.2. Stent Description

**Figure 1:**
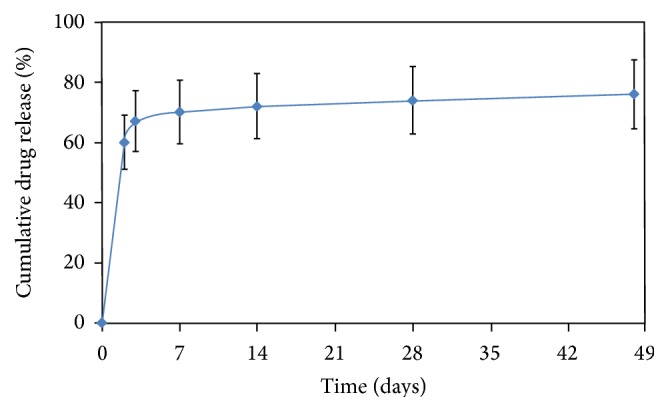
In vitro drug release from the Indolimus stent.

The Indolimus biodegradable polymer coated sirolimus-eluting coronary stent involves L605 cobalt chromium (Co-Cr) alloy as its stent platform. The biodegradable polymer gives it a strut thickness of 60 *μ*m and drug load of 1.4 *μ*g/mm^2^. About 70% of drug is released within 7 days and remaining drug is released over a period of 48 days (Figure 1). The drug is released within 7 weeks after the stent implantation from the polymeric layers coated onto the surface of the stent. The biodegradable polymeric film is a blend of different biodegradable polymers, poly L-lactide, 50/50 poly DL lactide-co-glycolide, and polyvinyl pyrrolidone, which undergoes hydrolysis. This process takes approximately 9 to 12 months after which all the polymers degrade naturally and excrete from body in the form of their metabolites.

The average coating thickness of Indolimus stent is between 5 and 6 *μ*m. The Indolimus stent is available in lengths of 8, 12, 16, 20, 24, 28, 32, 36, and 40 mm and available diameters were 2.5, 2.75, 3.0, and 3.5 mm.

### 2.3. Interventional Procedure and Adjunctive Medications

All patients received a loading dose of 300 mg of aspirin and clopidogrel (300 mg) or prasugrel (60 mg) or ticagrelor (90 gm). The procedural anticoagulation was achieved with either heparin or bivalirudin. However, the intraprocedural administration of glycoprotein IIb/IIIa-inhibitor was at the investigator's discretion. The procedure was performed according to the standard treatment guidelines of each participating centre. All the patients received dual antiplatelet therapy (aspirin 75–300 mg/daily indefinitely and clopidogrel 75 mg/daily or prasugrel 10 mg/daily or ticagrelor 90 mg twice daily for at least 6 months) after the procedure.

### 2.4. Study Endpoints

The primary endpoint of the study was a conglomeration of cardiac death, myocardial infarction (MI) (Q-wave and non-Q-wave), target lesion revascularization (TLR), target vessel revascularization (TVR), and stent thrombosis (ST). These endpoints were observed at 30-day, 6-month, and 9-month follow-up. The secondary endpoints will be measured at 12 and 24 months and yearly thereafter for five years.

### 2.5. Definition of Endpoints and Clinical Events

Procedural success was defined in terms of in-hospital MACE. MACE is composed of cardiac death, MI, TLR, or TVR. Death can be cardiac or noncardiac death. Any death due to undetermined cause was reported as cardiac death. Q-wave MI was considered, when there was development of new Q-wave of more than 0.04 seconds in two or more adjoining leads along with increase in cardiac markers like Troponin I or T, creatine kinase, or MB isoform. Non-Q-wave MI was considered when there was more than three-time elevation in creatinine kinase levels along with elevation in MB isoform and Troponin marker T or I without development of new Q-waves. Target lesion revascularization was considered when there was stenosis in treated segment (5 mm proximal and 5 mm distal edges) [11]. Target vessel revascularization was considered when there was stenosis in any segment of the treated vessel. Stent thrombosis (ST) was considered acute when it occurred within 24 hours, subacute when it occurred between 1 and 30 days, and late when it occurred after 30 days. The “definite” stent thrombosis was counted when it was detected angiographically.

### 2.6. Follow-Up

Clinical follow-up, by hospital appointment or telephonic conversation, was scheduled at 30 days (±7-day window period), 6 months (±15-day window period), and 9 months (±30-day window period). Follow-up data were collected pertaining to current anginal status, intake of antithrombotic regimen, and occurrence of any cardiovascular events or any invasive or noninvasive procedure that the patient had undergone.

## 3. Results

### 3.1. Baseline and Lesion Characteristics

A total of 372 diabetic patients with 437 lesions were treated with 483 SES. The average stent length and diameter were 27.1 ± 8.7 mm and 3.1 ± 0.4 mm, respectively. The baseline demographics of all the treated patients are described in Table 1. Out of all the diabetics (mean age = 53.4 ± 10.2 years), majority of them were male (68.8%). The prevalence of hypertension was seen in 202 (54.3%) patients. Double vessel disease was more prevalent and found in 111 (29.8%) patients. Lesions type B, type C and totally occluded lesions were found in 317 (72.5%), 51 (11.7%), and 45 (10.3%) patients, respectively. The detailed angiographic and procedural characteristics are described in Table 2.

### 3.2. Clinical Outcome

**Figure 2:**
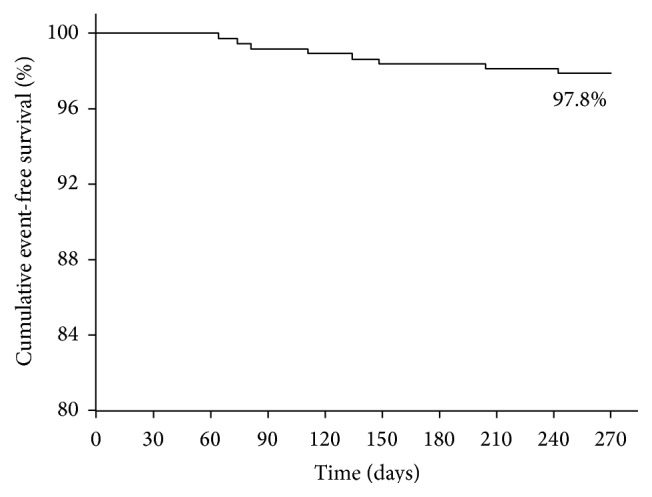
Cumulative event-free survival curve at 9-month follow-up.

The clinical follow-up at 9 months was obtained for 370 (99.4%) patients. At 9-month follow-up, MACE was found to be 2.2% which is a composite of 2 (0.5%) cases of myocardial infarction, 4 (1.1%) cases of target lesion revascularization, and 2 (0.5%) cases of stent thrombosis. The clinical outcomes of patients at 30-day, 6-month, and 9-month follow-up are shown in Table 3. The cumulative event-free survival by Kaplan Meier method was found to be 97.8% at 9-month follow-up (Figure 2).

## 4. Discussion

Does diabetes mellitus worsens the prognosis and long-term outcomes of patients with coronary artery disease? This long held question still poses a dilemma and the answer quite fluctuates between fact and fiction. It is long established that diabetes increases the rates of restenosis and repeat revascularization after coronary angioplasty [12, 13]. This belief was so severe that in the germinating era of bare metal stents surgery was considered as the primary option for patients with diabetes and multiple-vessel disease. The Bypass Angioplasty Revascularization Investigation (BARI) trial demonstrated the equivalence of angioplasty and bypass surgery but the five-year outcomes of BARI trial demonstrated crystal clear advantage of surgery in subset of patients with diabetes [2].

The introduction of drug eluting stents revolutionized the concept of safety and efficacy in diabetic population. The SIRIUS trial demonstrated that at 9 months TLR rate was reduced in diabetic population from 22.3% in BMS group to 6.9% in SES group [14]. The smaller nonrandomized Porto I trial also demonstrated TLR rates as low as 1.7% [15]. Consistent with this, our study also demonstrated a low TLR rate of only 1.1% at 9-month follow-up. The lower rates of restenosis associated with our stent can be because of lower strut thickness, as stents with lower stent thickness elicit less angiographic and clinical restenosis than stents with thicker struts [16].

Theoretically, due to aggressive atherosclerosis, platelet hyperactivity, impaired fibrinolysis, and endothelial function after arterial injury, diabetes mellitus is associated with antiplatelet resistance and consequent stent thrombosis after angioplasty. This has been proved in previous registry of DES [17]. But, paradoxically, our study presented only two cases of stent thrombosis at 9-month follow-up. This is also supported by the results from ISAR-DIABETES study and DIABETES trial [18, 19].

Elezi et al. reported that one-year event-free survival after stenting is lower in diabetics (73.1%) versus (78.8%) in nondiabetics [12]. The event-free survival at 9 months in our study was found to be 97.8%, which is quite appreciable. Contrastingly, there are studies which demonstrate that cumulative event-free survival is not affected by diabetic status [20].

The results of our study are quite promising. However the long-term follow-up of the study would prove maintained safety and efficacy.

## 5. Conclusions

The use of Indolimus in high risk diabetic population is associated with lower incidence of TLR, ST, and consequent MACE. Thus, the long held dilemma about the favorable outcomes after implantation of biodegradable polymer coated sirolimus-eluting coronary stent system in diabetic population turns out to be a fact and not fiction.

## Figures and Tables

**Table 1:** Baseline demographic characteristics.

Characteristics	Patients (*n* = 372 patients)
Age (mean ± SD, yrs)	53.4 ± 10.2
Male, *n* (%)	256 (68.8%)
Hypertension, *n* (%)	202 (54.3%)
Dyslipidemia, *n* (%)	24 (6.5%)
Family history of CAD, *n* (%)	17 (4.6%)
Smoking, *n* (%)	67 (18%)
Previous MI, *n* (%)	51 (13.7%)
Previous PCI, *n* (%)	65 (17.5%)
Previous CABG, *n* (%)	17 (4.6%)
Previous stroke, *n* (%)	8 (2.2%)

CAD: coronary artery disease, MI: myocardial infarction, PCI: percutaneous coronary intervention, and CABG: coronary artery bypass graft.

**Table 2:** Angiographic and procedural characteristics.

Characteristics	Patients (*n* = 372) Lesions = 437
Lesion location	
Right coronary artery, *n* (%)	140 (32.0%)
Left anterior descending, *n* (%)	195 (44.6%)
Left circumflex, *n* (%)	102 (23.3%)
Left marginal, *n* (%)	0 (0.0%)

ACC/AHA lesion classification	
Type A, *n* (%)	69 (15.8%)
Type B1, *n* (%)	167 (38.2%)
Type B2, *n* (%)	150 (34.3%)
Type C, *n* (%)	51 (11.7%)
Total occlusion, *n* (%)	45 (10.3%)

Number of diseased vessels	
Single vessel disease, *n* (%)	245 (65.9%)
Double vessel disease, *n* (%)	111 (29.8%)
Triple vessel disease, *n* (%)	16 (4.3%)

Procedural data	
Total number of stents, *n*	483
Number of stents per patient (mean ± SD, mm)	1.3 ± 0.5
Number of stents per lesion (mean ± SD, mm)	1.1 ± 0.3
Average stent diameter (mean ± SD, mm)	3.1 ± 0.4
Average stent length (mean ± SD, mm)	27.1 ± 8.7

ACC/AHA: American College of Cardiology/American Heart Association.

**Table 3:** Cumulative major adverse cardiac events at 30-day, 6-month, and 9-month follow-up.

Clinical outcomes	30-day follow-up	6-month follow-up	9-month follow-up
Death, *n* (%)	0 (0%)	0 (0%)	0 (0%)
Myocardial infarction, *n* (%)	0 (0%)	1 (0.3%)	2 (0.5%)
Q-wave, *n* (%)	0 (0%)	0 (0%)	1 (0.3%)
Non-Q-wave, *n* (%)	0 (0%)	1 (0.3%)	1 (0.3%)
Target lesion revascularization, *n* (%)	0 (0%)	3 (0.8%)	4 (1.1%)
Target vessel revascularization, *n* (%)	0 (0%)	0 (0%)	0 (0%)
Stent thrombosis, *n* (%)	0 (0%)	2 (0.5%)	2 (0.5%)
MACE, *n* (%)	0 (0%)	6 (1.6%)	8 (2.2%)
